# A Comparison of Endothelial Cell Loss in Combined Cataract and MIGS (Hydrus) Procedure to Phacoemulsification Alone: 6-Month Results

**DOI:** 10.1155/2015/769289

**Published:** 2015-11-17

**Authors:** Antonio M. Fea, Giulia Consolandi, Giulia Pignata, Paola Maria Loredana Cannizzo, Carlo Lavia, Filippo Billia, Teresa Rolle, Federico M. Grignolo

**Affiliations:** Department of Surgical Sciences, Eye Clinic, University of Turin, 10122 Turin, Italy

## Abstract

*Purpose*. To compare the corneal endothelial cell loss after phacoemulsification, alone or combined with microinvasive glaucoma surgery (MIGS), in nonglaucomatous versus primary open angle glaucoma (POAG) eyes affected by age-related cataract.* Methods*. 62 eyes of 62 patients were divided into group 1 (*n* = 25, affected by age-related cataract) and group 2 (*n* = 37, affected by age-related cataract and POAG). All patients underwent cataract surgery. Group 2 was divided into subgroups A (*n* = 19, cataract surgery alone) and B (*n* = 18, cataract surgery and MIGS). Prior to and 6 months after surgery the patients' endothelium was studied. Main outcomes were CD (cell density), SD (standard deviation), CV (coefficient of variation), and 6A (hexagonality coefficient) variations after surgeries.* Results*. There were no significant differences among the groups concerning preoperative endothelial parameters. The differences in CD before and after surgery were significant in all groups: 9.1% in group 1, 17.24% in group 2A, and 11.71% in group 2B. All endothelial parameters did not significantly change after surgery.* Conclusions*. Phacoemulsification determined a loss of endothelial cells in all groups. After surgery the change in endothelial parameters after MIGS was comparable to the ones of patients who underwent cataract surgery alone.

## 1. Introduction

The high rate of complications of traditional glaucoma surgery (trabeculectomy) has prompted the glaucoma community to search for alternative surgeries to treat primary open angle glaucoma (POAG) [1]. Recently, the interest for less invasive glaucoma surgeries has significantly increased. These new techniques have been collectively defined as microinvasive glaucoma surgery (MIGS). While tube surgery is generally reserved to cases where trabeculectomy is considered ineffective or has previously failed [2, 3], MIGS is recommended for initial to moderate glaucoma.

Most of the MIGS imply the insertion of a draining stent in the anterior chamber angle. The Hydrus microstent (Hydrus, Ivantis, Irvine, CA) is inserted into Schlemm's canal, bypassing the trabecular meshwork and providing direct aqueous access to Schlemm's canal.

It is known that the corneal endothelial cell density (EDC) progressively decreases over time [4, 5] and that any implant within the anterior chamber can result in progressive endothelial cell loss [4, 6–8]. Anterior chamber IOLs [9, 10] and tube surgery can determine endothelial damage: although the exact mechanism causing the damage after tube surgery is mostly unknown, the frequency of corneal complications after Ahmed Glaucoma Valve (AGV) implant surgery has been reported to be 27% after long-term follow-up [11].

Due to the often coexisting age-related cataract and to the easiness of a combined procedure, MIGS are best used in conjunction with cataract surgery, which can cause by itself damage to the corneal endothelium [12].

The effect of cataract surgery and MIGS implant on the corneal endothelium has not been previously investigated.

This prospective study compares the corneal endothelial cell loss after phacoemulsification in nonglaucomatous eyes to phacoemulsification in POAG eyes with and without combined MIGS implant over a six-month period.

## 2. Materials and Methods

This is a nonrandomised retrospective study on 62 consecutive patients, affected by uncomplicated age-related cataract. Patients have been divided into two groups: group 1 was affected by age-related cataract (*n* = 25) and group 2 presented age-related cataract and POAG (*n* = 37).

Patients were included if they had a diagnosis of age-related cataract, age between 55 and 84 years, corneal thickness (using ultrasound contact pachymetry) between 480 and 620 microns, absence of corneal dystrophies, central endothelial cell count of at least 1500 cells/mm^2^, no history of previous ocular surgery, no history of previous ocular inflammation/infection, and absence of other major eye diseases (diabetic retinopathy and age-related macular disease).

After full explanation of study procedures and signature of an informed consent, consecutive patients fulfilling these criteria and willing to participate to the study were recruited. The study protocol was approved by our local ethics committee.

Patients with a previous diagnosis of POAG confirmed by optic nerve evaluation, visual field, and a medicated IOP at the screening visit lower than 24 mmHg were placed in group 2. POAG subjects were excluded if they were using more than four medications or if they were on oral hypotensive drugs.

Patients in group 1 underwent cataract surgery alone, while patients in group 2 were randomly divided into two subgroups. Patients in subgroup 2A (*n* = 19) underwent cataract surgery alone, while those in subgroup 2B (*n* = 18) underwent a combined procedure (cataract surgery and Hydrus stent implantation). Patients characteristics are resumed in Table 1.

### 2.1. Surgical Procedure

All surgical procedures were performed by a single surgeon (AMF) according to the procedure described below. Cataract surgical times, total phacoemulsification times, and the percentage of ultrasound power for each procedure were recorded at the end of every intervention (Table 1).

All patients underwent cataract surgery with phacoemulsification through a 2.2 mm eleven o'clock clear corneal incision. After injection of a dispersive viscoelastic device (OVD) (Viscoat, Alcon laboratories, Fort Worth, TX), a 6 mm diameter capsulorhexis was carried out. Phacoemulsification and irrigation/aspiration of cortical material were performed using the same phacoemulsification instrument (AMO Signature White Star, Abbot, Chicago, IL). Then, a cohesive OVD (Provisc, Alcon) was injected in the capsular bag and a foldable single piece monofocal IOL was inserted in the capsular bag. The same IOL was implanted in all cases (Tecnis ZCB00, Abbott Laboratories Inc., Abbott Park, Illinois, USA). The OVD was completely removed from the anterior chamber (AC) and from the capsular bag using a monomanual irrigation-aspiration system.

After phacoemulsification, the patients who underwent combined surgery (group 2B) had the microscope repositioned and the head tilted to allow a clear view of the angle structures with a gonioprism. Additional viscoelastic device (Healon GV, Abbott) was introduced for chamber maintenance and optimum view. The Hydrus delivery cannula was inserted through a 1–1.5 mm secondary temporal incision. The beveled tip of the cannula was used to perforate the trabecular meshwork and the microstent was implanted into Schlemm's canal by advancing the tracking wheel with the index finger, leaving 1-2 mm (the inlet segment) in the anterior chamber in the nasal quadrant. Upon gonioscopic confirmation of position in the canal, the delivery system was withdrawn and viscoelastic device removed; the AC was inflated with balanced salt solution to achieve normal IOP.

At the end of surgery the eye was inflated through the accessory wound. No sutures were needed to seal the wound.

Postoperative care included a topical antibiotic for 4–7 days and a tapering dose of a topical corticosteroid for 4 weeks in all groups.

### 2.2. Endothelium Analysis

The endothelium was studied using the proprietary software of the Konan Cell Check XL (Konan Medical, Irvine, CA, USA). CD (cell density), SD (standard deviation), CV (coefficient of variation), and 6A (hexagonality coefficient) were evaluated. The endothelium was examined before intervention and 6 months after surgery. The examinations were performed by an expert operator (CAL) and endothelial cell data were based on the average of the three measurements in the central cornea with the best clarity at each site. Images of low quality were excluded and the examination was repeated until they were clear enough. If it was not possible, the patients were excluded from the study. Endothelial cells were analyzed using the dot method, in which the sites of approximately 80–100 contiguous cells were marked. One glaucoma specialist (AMF) interpreted all endothelial cell data. Endothelial damage was defined as the difference between the preoperative and postoperative values.

### 2.3. Statistical Analysis

One sample *t*-test was performed for data comparison within groups and two-sample *t*-test for data comparison between groups. Statistical analysis was performed using Analyse-it statistical software for Microsoft Excel (version 2.26; Analyse-it software, Leeds, UK). The limit of statistical significance was set at *p* ≤ 0.05.

## 3. Results

Preoperative data regarding the patients are reported in Table 1. There were no significant differences between the groups for age and cataract grade (LOCS III) [13].

Preoperatively, the mean CD was 2361.7 ± 477.9 cells/mm^2^ in group 1, 2234.5 ± 344.7 cells/mm^2^ in group 2A, and 2476.2 ± 300.6 cells/mm^2^ in group 2B (Table 1). There were no statistically significant differences between groups.

There were no differences between the groups concerning phacoemulsification times and perceptual US powers (Table 1). As expected, the total surgical time of the combo surgery was longer than cataract surgery only (group 2B versus group 1: *t* = 8.2, *p* < 0.001; group 2B versus group 2A: *t* = 8.6, *p* < 0.001).

We observed a significant loss of endothelial cells following all procedures (Table 2).

The corneal CD decreased significantly in all groups after phacoemulsification, with a mean CD reduction of 214.4 cells/mm^2^ in group 1 (2361.7 ± 477.9 to 2147 ± 455.7 cells/mm^2^; *p* < 0.01), 362.1 cells/mm^2^ in group 2A (2234.5 ± 344.7 to 1872 ± 393.5 cells/mm^2^; *p* < 0.01), and 290.3 cells/mm^2^ in group 2B (2476.2 ± 300.6 to 2185.8 ± 393.1 cells/mm^2^; *p* < 0.01).

The SD and CV did not differ significantly before and after surgery in any group.

Finally, the 6A changed significantly after phacoemulsification for age-related cataract (group 1: 57.6 ± 10.5 to 51.4 ± 10.1; *p* = 0.015) and phacoemulsification + stent in patients with POAG (group 2B: 56.3 ± 7.5 to 62.5 ± 10; *p* = 0.016), though not for POAG treated with phacoemulsification alone (group 2A: 55.5 ± 13.1 to 55.9 ± 8.9; *p* = 0.865).

In Table 2 the difference between the pre- and postoperative data was reported for the three groups and in Table 3 the results of the statistical analysis of the same data are shown. Within the three groups, no significant differences between pre- and postoperative data were found.

## 4. Discussion

The new ab interno glaucoma surgeries through a clear corneal incision (MIGS) aim at reducing the potential complications of traditional glaucoma surgery. They present several other advantages: the sparing of the conjunctiva, allowing future glaucoma surgery if needed, direct visualization of anatomic landmarks, and maintenance of the anterior chamber with negligible disruption of normal anatomy and physiology [1].

These new less invasive surgical approaches can easily be combined with cataract surgery and several studies proved their efficacy in reducing the IOP [14–17]. Nevertheless, all the new trabecular shunting devices need to be inserted passing through the anterior chamber and lie in the chamber angle very close to the corneal endothelium. Although all these procedures avoid the common complications of trabecular surgery, they might determine a progressive loss of endothelial cells.

We analyzed the effect of the placement of the Hydrus stent in patients undergoing combined surgery and we compared our results with a group of patients with and without glaucoma after uncomplicated cataract surgery, to avoid the confounding factor of the damage induced by the phacoemulsification itself. To our knowledge, there is no previous report which analyzes corneal endothelial cell loss after combined surgery with MIGS implant compared to phacoemulsification alone.

Phacoemulsification determined a loss of endothelial cells in all groups. Our results are in line with what was reported by other authors (Table 4). The phacoemulsification parameters between the three groups were similar as was the degree of cataract. Although the total surgical time was longer in the combo group, we did not observe any further damage in this group of patients. At six months the change in the endothelial parameters after implantation of the Hydrus stent was comparable to the ones of patients who underwent cataract surgery. This is extremely important because it is well known that any device in the anterior chamber can cause some degree of damage [2, 18–20]. MIGS have been developed to address patients with mild to moderate glaucoma and any damage to the corneal endothelium would be considered a serious adverse event.

Our study presents some limitations: the number of patients is relatively small (but we must consider that this is a relatively new procedure), the follow-up is relatively short, and there is just one postoperative follow-up. Further data need to be gathered in the future.

## Figures and Tables

**Table 1:** Preoperative demographic and operative data of the patients (mean ± standard deviation; LOCS: lens opacity classification system; CD: cell density; SD: standard deviation; CV: coefficient of variation; 6A: hexagonality coefficient; US: ultrasound; *p*: *p* value; N/A: not applicable).

Group	1	2A	2B	*p* 1 versus 2A	*p* 1 versus 2B	*p* 2A versus 2B
Mean age (yrs)	70.3 ± 2.5	68.8 ± 2.7	69.6 ± 2.2	0.064	0.347	0.332
Gender m/f (%)	17 (68)/8 (32)	9 (47.4)/10 (52.6)	15 (82.4)/4 (17.6)	N/A	N/A	N/A
Cataract grade (LOCS III)	3.71 ± 0.9	3.4 ± 0.8	3.64 ± 1.2	0.242	0.828	0.477
CD (cell/mm^2^)	2361.7 ± 477.9	2234.5 ± 344.7	2476.2 ± 300.6	0.332	0.376	0.052
SD (*μ*m^2^)	171.8 ± 84.2	173.8 ± 63.4	155.5 ± 41.9	0.932	0.455	0.31
CV	37.6 ± 9.8	36.9 ± 9.8	37.3 ± 8.2	0.816	0.916	0.894
6A (%)	57.6 ± 10.5	55.5 ± 13.1	56.3 ± 7.5	0.558	0.656	0.822
Total surgical time (mins)	12.3 ± 2.5	12 ± 1.9	18.45 ± 2.9	0.665	<0.001	<0.001
Phacoemulsification time (sec)	37.1 ± 19.7	34 ± 18.4	35.4 ± 17	0.598	0.769	0.812
US power (%)	20.8 ± 12	18.9 ± 6.1	20.5 ± 10.3	0.532	0.932	0.567

**Table 2:** Difference and statistical analysis between preoperative and postoperative parameters (mean ± standard deviation; CD: cell density; SD: standard deviation; CV: coefficient of variation; 6A = hexagonality coefficient).

Group	Parameters	CD	SD	CV	6A
1	Preoperative	2361.7 ± 477.9	171.8 ± 84.2	37.6 ± 9.8	57.6 ± 10.5
	Postoperative	2147.3 ± 455.7	178.7 ± 63.9	38.1 ± 8.7	51.4 ± 10.1
	Difference	−214.4 ± 362.6	6.92 ± 77.1	0.52 ± 10.2	−6.24 ± 11.9
	*t*-test	2.956	0.449	0.256	2.607
	*p* value	0.007	0.658	0.800	0.015

2A	Preoperative	2234.5 ± 344.7	173.8 ± 63.4	36.9 ± 9.8	55.5 ± 13.1
	Postoperative	1872.4 ± 393.5	216.7 ± 66.3	37.8 ± 8.1	55.9 ± 8.9
	Difference	−362.1 ± 316	42.9 ± 71.2	0.95 ± 11.1	0.47 ± 11.9
	*t*-test	4.995	2.63	0.373	0.173
	*p* value	<0.001	0.017	0.713	0.865

2B	Preoperative	2476.2 ± 300.6	155.5 ± 41.9	37.3 ± 8.2	56.3 ± 7.5
	Postoperative	2185.8 ± 393.1	170.8 ± 50.1	36.2 ± 7.5	62.5 ± 10
	Difference	−290.3 ± 322.4	15.3 ± 62.6	−1.1 ± 12	6.2 ± 12.2
	*t*-test	4.592	1.25	0.459	2.573
	*p* value	<0.001	0.224	0.65	0.016

**Table 3:** Statistical analysis of the differences between pre- and postoperative data for the three groups (CD: cell density; SD: standard deviation; CV: coefficient of variation; 6A: hexagonality coefficient).

Group	Parameters	CD	SD	CV	6A
1 versus 2A	*t*-test	1.306	1.587	0.133	1.846
	*p* value	0.199	0.12	0.895	0.072

1 versus 2B	*t*-test	0.792	0.427	0.513	3.661
	*p* value	0.432	0.671	0.610	0.001

2A versus 2B	*t*-test	0.743	1.38	0.579	1.557
	*p* value	0.461	0.175	0.566	0.127

**Table 4:** Reported mean endothelial cell loss after cataract surgery (SICS: small incision cataract surgery; ECD: endothelial cell density) [20–25].

Author	Year	Technique	Mean ECD loss (%)
Gogate et al. [20]	2010	Phacoemulsification	18.4
		SICS	17.7

Reuschel et al. [22]	2010	Phacoemulsification	7.2

Mathew et al. [23]	2011	Phacoemulsification	16.6

Tsuneoka et al. [21]	2002	Phacoemulsification	7.8

Walkow et al. [25]	2000	Phacoemulsification	8.5

Ataş et al. [24]	2014	Phacoemulsification	6.41

Present study	2015	Phacoemulsification	12.2
		Phacoemulsification + stent	11.71
